# Supplementary material for: pH-Sensitive Silver-Containing Carbon Dots Based on Folic Acid
Source: Materials (Basel). 2022 Mar 3;15(5):1880. doi: 10.3390/ma15051880 (PMC8912051; doi:10.3390/ma15051880)
Supplement: Supplementary file 1 [file materials-15-01880-s001.zip › materials-1581802-supplementary.pdf]

materials

MDPI

Supplementary Materials

pH-Sensitive Silver-Containing Carbon Dots Based on Folic Acid

Qinhai Xu, Kang Li and Peng Wang *

S1.1. Reagents

Folic acid (FA, $C_{19}H_{19}N_7O_6$), sliver nitrate ($AgNO_3$), Iron sulfate heptahydrate ($FeSO_4 \cdot 7H_2O$), Cobalt chloride hexahydrate ($CoCl_2 \cdot 6H_2O$), Manganese chloride ($MnCl_2 \cdot 4H_2O$) and Nickel sulfate hexahydrate ($NiSO_4 \cdot 6H_2O$) were obatained from Aladdin Industrial Corporation, (Shanghai, China). Barium Chloride Dihydrate ($BaCl_2 \cdot 6H_2O$) and Quinine sulfate dihydrate were obtained from Shanghai Titan Scientific Co., Ltd., (Shanghai, China). Calcium chloride anhydrous ($CaCl_2$), Sulfuric acid (H_2SO_4, A.R. 98%), Sodium nitrate ($NaNO_3$) and Sodium hydroxide (NaOH) were obtained from Sinopharm Chemical Reagent Beijing Co., Ltd., (Beijing, China). Chromic chloride hexahydrate ($CrCl_3 \cdot 6H_2O$) was obtained from Adamas Reagent Co., Ltd., (Shanghai, China). Magnesium chloride hexahydrate ($MgCl_2 \cdot 6H_2O$), Zinc Chloride ($ZnCl_2$) and Potassium chloride (KCl) were obtained from Beijing Chemical Works (Beijing, China). Cupric sulfate, anhydrous ($CuSO_4$) was obtained from Beijing Yili Chemical Co., Ltd., (Beijing, China). Lead Chloride ($PbCl_2$) was obtained from Tianjin KERMEL Chemical Reagents Development Centre (Tianjin, China). The Ba^{2+}, Ca^{2+}, Co^{2+}, Cr^{3+}, Cu^{2+}, Fe^{2+}, K^+, Mg^{2+}, Mn^{2+}, Na^+, Ni^{2+}, Pb^{2+} and Zn^{2+} solutions (1mM) were prepared in ultra-pure water from the salts of $BaCl_2 \cdot 6H_2O$, $CaCl_2$, $CoCl_2 \cdot 6H_2O$, $CrCl_3 \cdot 6H_2O$, $CuSO_4$, $FeSO_4 \cdot 7H_2O$, KCl, $MgCl_2 \cdot 6H_2O$, $MnCl_2 \cdot 4H_2O$, $NaNO_3$, $NiSO_4 \cdot 6H_2O$, $PbCl_2$ and $ZnCl_2$, respectively. All chemicals were analytical grade and all solutions were prepared by ultra-pure water at room temperature.

S1.2. Apparatus

Transmission electron microscopy (TEM) was obtained by a TECNAI transmission electron microscope operated at 300 kV. The elemental analysis of Ag-CQDs was measured by X-ray photoelectron spectroscopy (XPS) on a Thermo ESCALAB 250Xi spectrometer (Setting parameters: monochromatic Al Ka (hv = 1486.6 eV), operated at 150 W, 500 μm beam spot, USA). Fourier-transformed infrared spectroscopy (FTIR) was obtained by a IRPrestige-21 (SHIMADZU, CaF wafer technique, Japan). UV-Vis spectrum was recorded on a UV-3600 UV-VIS-NIR spectrophotometer (SHIMADZU, Japan). The fluorescence spectra were taken on a fluorescence Spectrophotometer (HITACHI F-4600, Japan). The pH values of solutions were measured by a digital pH meter (PHS-3C, YOKE INSTRUMENT, China). Ultra-pure water was obtained from a Millipore Mingche™-D 24^{UV} water purification system.

S1.3. XPS Spectrum of Ag-CDs

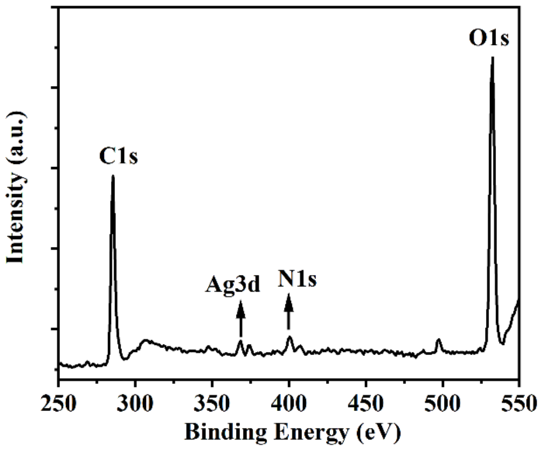

Figure S1. XPS spectrum of Ag-CDs.

S1.4. Photo-Stability Evaluation

The photo-stability of Ag-CDswas evaluated by the following method. Under excitation at 360 nm, the fluorescence intensity probed at 400 and 450 nm, respectively, were measured continuously for 180 seconds. The results were shown as Figure S2, which indicated that the fluorescence intensities decreased by 4.42% at 400 nm and 2.48% at 450 nm, respectively. This result proved that Ag-CDs prepared in this research has good photo-stability [1,2].

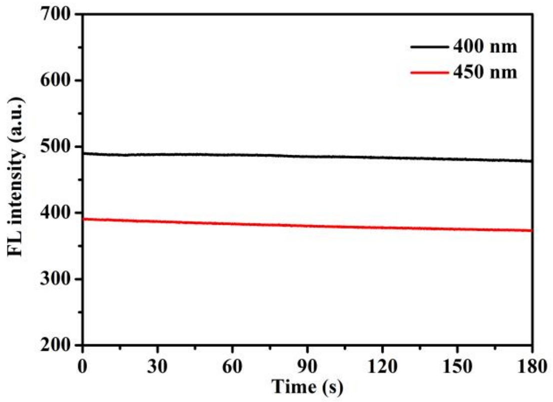

Figure S2. The photo-stability test. The fluorescence intensities of Ag-CDs which were continuously measured for 180 seconds at 400 and 450 nm under excitation at 360 nm.

S1.5. Quantum Yield (QY) Measurements

The quantum yield of Ag-containing carbon dots (Ag-CDs) was measured by using a reference method, comparing the integrated fluorescence intensity (excited at 365 nm) and the absorbance (less than 0.1 at the excitation wavelength) of Ag-CDs solutions with those of the standards. Quinine sulfate dissolved in 0.1 M H_2SO_4 solution (quantum yield of 53.0% excited at 365nm) was selected as a criterion by the following equation:

$$\Phi = \Phi_S[(I/I_S)\ (A_S/A)\ (n^2/n_S^2)] \quad (S1)$$

where "Φ" is the quantum yield, "I" is the fluorescence peak area at the maximum excitation wavelength, the subscript "S" represents the reference quinine sulfate, "A" is the UV-Vis absorbance intensity, "n" is the different refractive index of solvent, the superscript "2" means the square of "n".

By selecting quinine sulfate as a criterion, the quantum yield (QY) of Ag-CDs was calculated to be 14.43% according to Equation (S1).

S1.6. The Effect of pH on the Fluorescence of Ag-CQDs

It could be seen from Figure S3, the changes in the fluorescence of Ag-CQDs related to pH (10.20-13.95) were observed. The fluorescence spectra of Ag-CQDs at pH (10.20-11.35) had no change. When the pH were more than or equal to 11.55, the fluorescence intensities of Ag-CQDs significantly enhanced. The pH was 11.75, the fluorescence intensity of Ag-CQDs reached the maximum. Then, when the pH were more than 11.75, the fluorescence intensities of Ag-CQDs decreased continuously. Therefore, Ag-CQDs could be used as a pH fluorescent probe based on the change of the fluorescence for environment monitoring applications under extremely alkaline conditions.

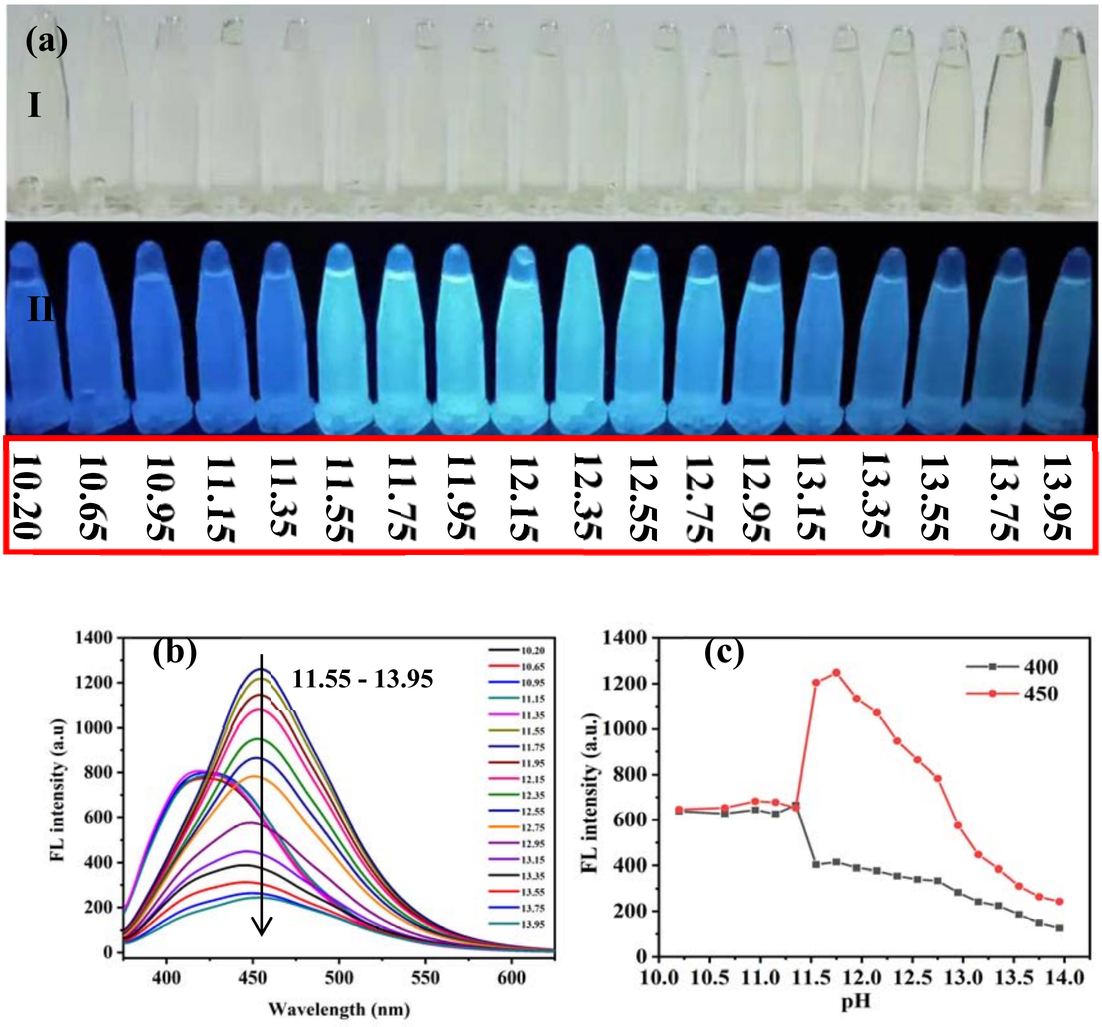

Figure S3. (**a**) Photographs of Ag-CQDs at different pH (10.20 - 13.95) under daylight (I) and UV light (II, excited at 365 nm). (**b**) The fluorescence spectrum of Ag-CQDs at different pH, λex = 360 nm, respectively. (**c**) The fluorescence intensities at 400 and 450 nm of Ag-CQDs at different pH, λex = 360 nm, respectively.

S1.7. The Fluorescence Lifetime of Ag-CQDs

The fluorescence lifetime of Ag-CDs was further measured by time correlated single photon counting (TCSPC) method. The fluorescence lifetime of Ag-CDs was obtained by an Edinburgh FLS980 all functional steady-state/transient fluorescence spectrometer (Edinburgh Instruments, UK). The fluorescence decay curve of the dual-emission Ag-CDs was fitted to a two-exponential function:

$$R(t) = 198.009 * e^{(-\tau 1/2.8608)} + 171.866 * e^{(-\tau 2/9.1259)} \quad \text{(S2)}$$

As shown in Figure S4, the fluorescence lifetime (τ_1/τ_2) of Ag-CDs was 2.8608 (26.53%)/9.1259 (73.47%) ns, respectively, and the average lifetime was calculated be 7.4638 ns.

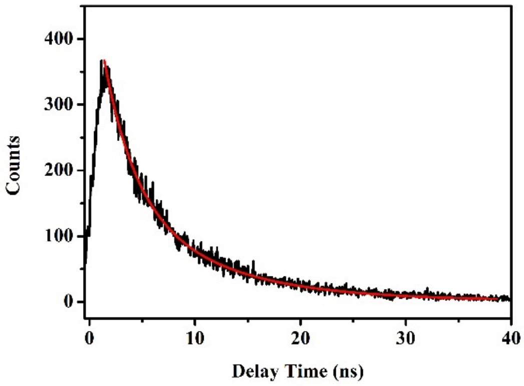

Figure S4. The fluorescence lifetime of Ag-CDs.

S1.8. Fluorescence Stability of Ag-CQDs

To evaluate the effect of metal ions on the fluorescence of Ag-CQDs, 4 mL of various metal cations (Ba^{2+}, Ca^{2+}, Co^{2+}, Cr^{3+}, Cu^{2+}, Fe^{2+}, K^{2+}, Mg^{2+}, Mn^{2+}, Na^{2+}, Ni^{2+}, Pb^{2+} and Zn^{2+}, 1 mM) were respectively added into 13 parts of 0.5 mL of Ag-CQDs solutions. The fluorescence emission spectra were recorded by the fluorescence spectrophotometer (HITACHI F-4600). As shown in Figure S5a–c, when cations (Ba^{2+}, Ca^{2+}, Co^{2+}, Cr^{3+}, Cu^{2+}, Fe^{2+}, K^{+}, Mg^{2+}, Mn^{2+}, Na^{+}, Ni^{2+}, Pb^{2+} and Zn^{2+}, 1 mM) were added into Ag-CQDs solutions, respectively. The photographs and normalized fluorescence intensity of Ag-CQDs containing cations exhibited that most of these metal ions had little influence on the fluorescence of Ag-CQDs, which could be negligible, illustrating the good ions-stability of Ag-CQDs.

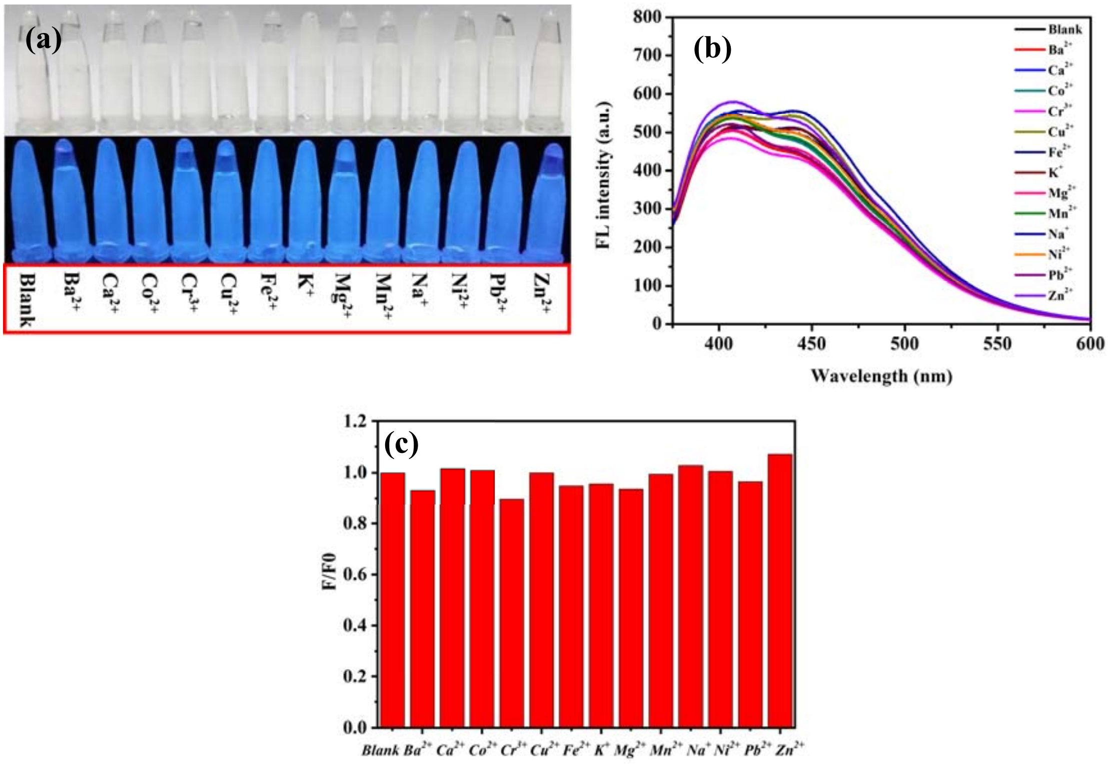

Figure S5. (**a**) Photographs of Ag-CQDs containing metal ions (Ba^{2+}, Ca^{2+}, Co^{2+}, Cr^{3+}, Cu^{2+}, Fe^{2+}, K^+, Mg^{2+}, Mn^{2+}, Na^+, Ni^{2+}, Pb^{2+} and Zn^{2+}, 1 mM) under daylight (up) and UV light (down, excited at 365 nm). Fluorescence emission spectra (**b**) and normalized fluorescence intensities (**c**) of Ag-CQDs containing metal ions (Ba^{2+}, Ca^{2+}, Co^{2+}, Cr^{3+}, Cu^{2+}, Fe^{2+}, K^+, Mg^{2+}, Mn^{2+}, Na^+, Ni^{2+}, Pb^{2+} and Zn^{2+}, 1 mM), λex = 360 nm. .

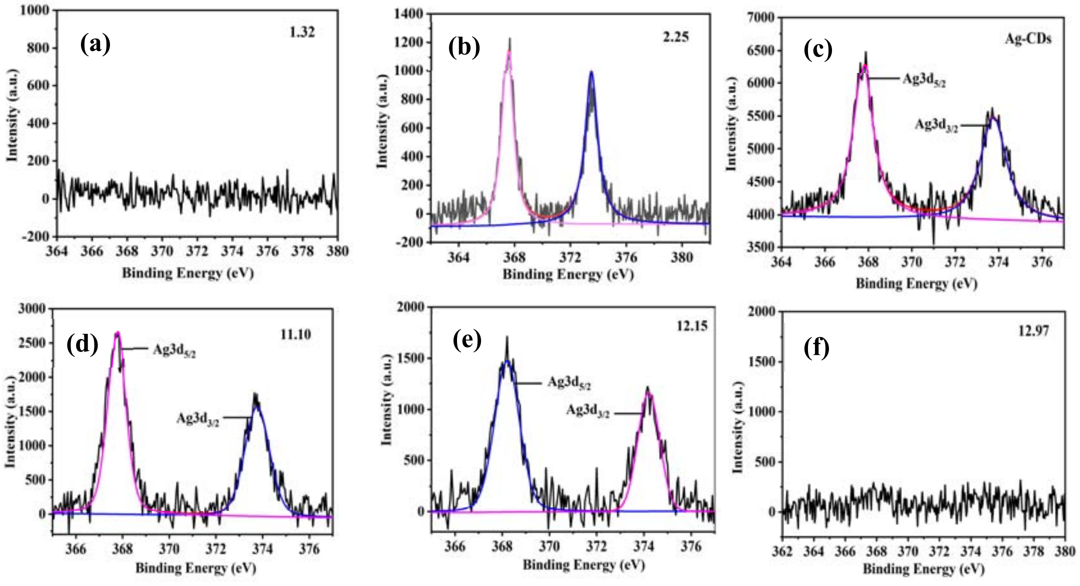

Figure S6. High-resolution XPS spectra of Ag3d of Ag-CDs at pH 1.32 (**a**), 2.25 (**b**), Ag-CDs (**c**), 11.10 (**d**), 12.15 (**e**), 12.97 (**f**), respectively.

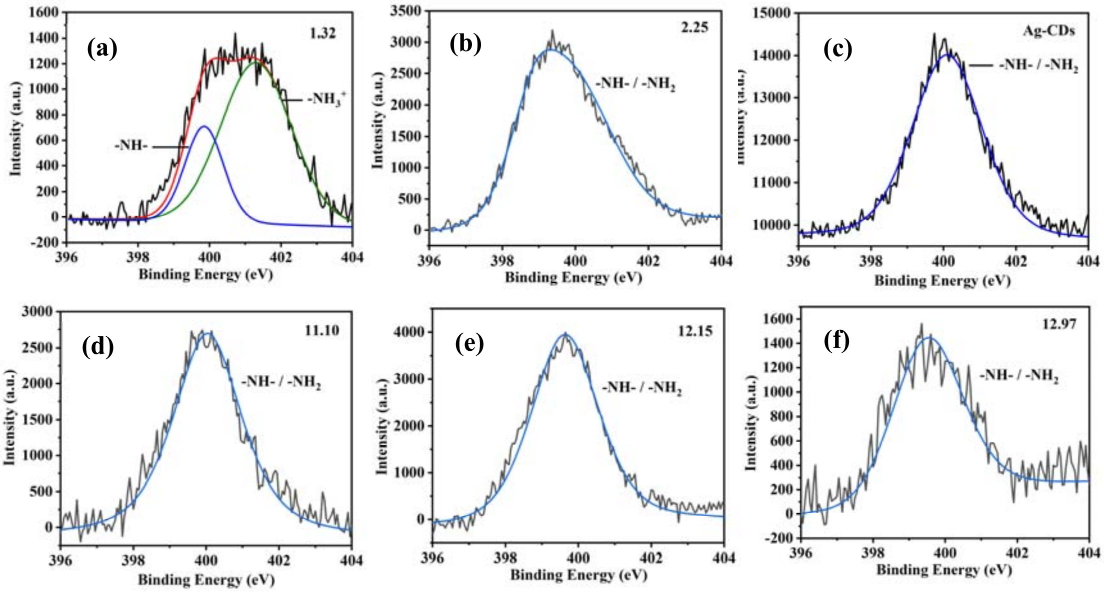

Figure S7. High-resolution XPS spectra of N1s of Ag-CDs at pH 1.32 (**a**), 2.25 (**b**), Ag-CDs (**c**), 11.10 (**d**), 12.15 (**e**), 12.97 (**f**) , respectively.

Table S1. Comparisons of pH sensing range of various fluorescent probes based on carbon dots.

Detection Probes	pH Range	Ref.
Carbon dot	5.2 to 8.8	Shangguan et al. 2016 [3]
Graphene quantum dots	3 to 9	Hai et al. 2015 [4]
Carbon dots	3 to 9	Wang et al.2015 [5]
Carbon dots	5 to 8	Nie et al. 2014 [6]
Carbon dots	4 to 8	Du et al. 2013 [7]
N-CQDs	2.87 to 7.24	Liao et al. 2018 [8]
Carbon dots	pH 5 to 10	Sun et al. 2018 [9]
Carbon dots	4.0–7.5 and 7.5–13.0	Zhi Ma et al. 2020 [10]
Carbon dots	1.05 to 10.97	Chuanxi Wang et al. 2015 [11]
Ag-CDs	≥12 and ≤1.32	This work

References

1. Mondal, T.K.; Saha, S.K. Facile Approach To Synthesize Nitrogen- and Oxygen-Rich Carbon Quantum Dots for pH Sensor, Fluorescent Indicator, and Invisible Ink Applications. ACS Sustain. *Chem. Eng.* **2019**, *7*, 19669–19678, https://doi.org/10.1021/acssuschemeng.9b04817.
2. Shekarbeygi, Z.; Karami, C.; Esmaeili, E.; Moradi, S.; Shahlaei, M. Development of Ag nanoparticle-carbon quan-tum dot nanocomplex as fluorescence sensor for determination of gemcitabine. *Spectrochim. Acta Part A Mol. Biomol. Spectrosc.* **2021**, *262*, 120148.
3. Shangguan, J.-F.; He, D.-G.; He, X.-X.; Wang, K.-M.; Xu, F.-Z.; Liu, J.-Q.; Tang, J.-L.; Yang, X.; Huang, J. Label-free carbon-dots-based ratiometric fluorescence pH nanoprobes for intracellular pH sensing. *Anal. Chem.* **2016**, *88*, 7837–7843.
4. Hai, X.; Wang, Y.-T.; Hao, X.-Y.; Wang, J.-H. Folic acid encapsulated graphene quantum dots for ratiometric pH sensing and specific multicolor imaging in living cells. *Sens. Actuators B Chem.* **2018**, *268*, 61–69.
5. Wang, C.-X.; Xu, Z.-Z.; Cheng, H.; Lin, H.-H.; Humphrey, M.-G.; Zhang, C. A hydrothermal route to water-stable luminescent carbon dots as nanosensors for pH and temperature. *Carbon* **2015**, *82*, 87–95.
6. Nie, H.; Li, M.-J.; Li, Q.-S.; Liang, S.-J.; Yan, Y.-Y.; Sheng, L.; Shi, W.; Zhang, S.-X. Carbon dots with continuously tunable full-color emission and their application in ratiometric pH sensing. *Chem. Mater.* **2014**, *26*, 3104–3112.
7. Du, F.-K.; Ming, Y.H.; Zeng, F.; Yu, C.-G.; Wu, S.-Z. A low cytotoxic and ratiometric fluorescent nanosensor based on carbon-dots for intracellular pH sensing and mapping. *Nanotechnology* **2013**, *24*, 365101.
8. Liao, S. Huang, X.-Q. Yang, H.; Chen, X.-Q. Nitrogen-doped carbon quantum dots as a fluorescent probe to detect copper ions, glutathione, and intracellular pH. *Anal. Bioanal. Chem.* **2018**, *410*, 7701–7710.

9. Sun, Y.-Q.; Wang, X.-J.; Wang, C.; Tong, D.-Y.; Wu, Q.; Jiang, K.-L.; Jiang, Y.-N; Wang, C.-X.; Yang, M.-H. Red emitting and highly stable carbon dots with dual response to pH values and ferric ions. *Microchim. Acta* **2018**, *185*, 83.
10. Ma, Z.; Ma, Y.; Gu, M.-Y.; Huo, X.-Y.; Ma, S.-N.; Lu, Y.-N.; Ning, Y.; Zhang, X.; Tian, B.; Feng, Z.-B. Carbon Dots Derived from the Maillard Reaction for pH Sensors and Cr (VI) Detection. *Nanomaterials* **2020**, *10*, 1924.
11. Wang, C.-X.; Xu, Z.-Z.; Zhang, C. Polyethyleneimine-Functionalized Fluorescent Carbon Dots: Water Stability, pH Sensing, and Cellular Imaging. *ChemNanoMat* **2015**, *1*, 122–127.
